# Adolescents’ engagement in multiple risk behaviours is associated with concussion

**DOI:** 10.1186/s40621-020-0233-8

**Published:** 2020-02-17

**Authors:** Joshua Shore, Ian Janssen

**Affiliations:** 1 School of Kinesiology and Health Studies, Queen’s University, 28 Division Street, Kingston, Ontario K7L 3N6 Canada; 2 Department of Public Health Sciences, Queen’s University, Kingston, Canada

**Keywords:** Multiple risk behaviours, Concussion, Injury, Epidemiology, Adolescent

## Abstract

**Background:**

The objective of this study was to investigate the relationship between engagement in multiple risk behaviours (MRB) and concussion amongst youth.

**Methods:**

This was a cross-sectional study that used survey data collected from 3059 students in grades 6–10 (approximate ages 11–15 years) from Ontario, Canada. Students reported whether or not they had a medically diagnosed concussion within the previous 12 months and the frequency that they participated in several risky behaviours including fighting, bullying, smoking, drinking alcohol, using illicit drugs, drinking caffeinated beverages, not using protective equipment, and having unsafe sex. Responses to the risky behavior items were used to create a MRB score. The association between MRB and concussion was explored using logistic regression that controlled for several confounding variables.

**Results:**

Approximately 10.7% of students reported that they had a medically diagnosed concussion within the past year. A dose-response relationship was found between MRB and concussion among students in grades 9–10, while in grades 6–8 students only those in the highest MRB quartile had an increased likelihood of concussion. The relative odds for concussion in the highest versus the lowest MRB quartile were 4.67 (95% confidence interval: 2.33, 9.35) in grades 9–10 students and 2.94 (95% confidence interval, 1.90, 4.56) in grades 6–8 students.

**Conclusions:**

Engagement in MRB may be an important etiologic component of adolescent concussion. Future studies should address whether behavioural interventions designed to decrease engagement in MRB reduce the risk of concussion and other injuries.

## Background

Traumatic brain injury is a major trauma-related cause of death and disability (Rubiano et al., 2015). Most traumatic brain injuries are of a mild severity and are commonly referred to as concussion (Dewan et al., 2018). Adolescents are more sensitive to the neurophysiological consequences of concussions than are adults (Baillargeon et al., 2012).

Recently, there has been a surge in diagnosed concussion rates. For instance, in Canada the annual incidence of medically diagnosed pediatric concussion increased from 34 per 10,000 persons in 2003 to 150 per 10,000 persons in 2013 (Zemek et al., 2017). There has also been an increase in research on the physiological mechanisms and causes of concussion among people of all ages (Signoretti et al., 2011). Sport participation is implicated in the etiology of about half of all pediatric concussions (Gordon et al., 2006). Motor vehicle accidents and falls are next leading causes of pediatric concussions (Gordon et al., 2006). Although the activities leading to concussion are well-established, a paucity of research has examined the social and behavioural factors, such as engagement in multiple risk behaviours (MRB), that may identify young people at heightened concussion risk.

MRB, such as alcohol consumption, drug use, and unprotected sex represent a clustering of behaviours that often develop together during adolescence and which indicate an increased tendency for risk-taking (Dryfoos, 1991). Engagement in MRB represents an adolescent’s need for novel and varied sensation seeking experiences, and is an indicator of their willingness to take social and physical risks (Zuckerman, 1979). Adolescents who take physical risks may increase their injury risk. Indeed, studies of demographically diverse adolescent samples report that MRB are associated with the occurrence of general and site specific injury (Koven et al., 2005; Pickett et al., 2002a; Pickett et al., 2002b). Studies have also reported that engagement in specific risky behaviours, such as alcohol use (Alcock et al., 2018) and physical violence (Buckley and Chapman, 2017), are associated with concussion. However, to our knowledge, the association between MRB and concussion has not been examined.

Therefore, our objective was to investigate the relationship between engagement in MRB and concussion among adolescents. We hypothesized that adolescents engaging in more MRBs would be more likely to have a concussion.

## Methods

### Study population and design

Study data are from the 2013/2014 Canadian Health Behaviour in School-Aged Children (HBSC) study (Freeman, 2016). The HBSC is a nationally representative survey. It was comprised of a confidential classroom-based health questionnaire completed by grade 6–10 students (approximate ages 11–15 years). A cluster sample design was used with school classes as the basic cluster. Classes were selected across all provinces in territories using a probability technique to ensure proportional representation by community size and location, language, and religion.

This study only included data from Ontario participants, as the surveys administered in other provinces and territories did not assess concussions. The Ontario sample consisted of 5888 students from 81 schools. We excluded 2838 students who did not respond to the concussion item, one or more of the MRB items, or the confounding variables, leaving a final sample of 3050.

## Concussion

The concussion item asked: “During the past 12 months, have you been told by a doctor or nurse that you had a concussion?”. Students who responded to the “Yes, 1 time” or “Yes, more than 1 time” options were placed into the concussion group. Injury reporting patterns in the HBSC accurately reflect injuries treated in the emergency room (Pickett et al., 2000).

### Multiple risk behaviours

MRB assessment was based on a composite MRB indicator developed in the HBSC (Kwong et al., 2018). Thirteen risky behaviours were considered: bullying, physical fighting, cigarette smoking, use of alternative tobacco products, alcohol consumption (frequency of drinking, number of drinks per typical event, drunkenness, binge drinking), cannabis use, illicit drug use, sexual history, helmet non-use on a bicycle, and caffeinated beverage consumption. Survey items instructed participants to report on their frequency of engagement using ordinal response categories. As explained in Table 1, responses were recoded into 3 categories describing no, moderate, or high engagement (Kwong et al., 2018). These recoded variables were included in a factor analysis, separately for grade 6–8 and 9–10 students, to derive a composite MRB score. A single factor emerged within each grade group. Students were placed into quartiles based on their factor score. Separate factor analyses were performed in grade 6–8 and 9–10 students because the more sensitive MRB items on drug use and sexual intercourse were not included in the grade 6–8 survey.

**Table 1:** List of risky behaviours and criteria used to define level of engagement

Risky behaviour	No engagement	Moderate engagement	High engagement
Frequency of bullying	None	1–3 times a month	≥ 1 a week
Physical fights in past year	None	1 fight	≥ 2 fights
Lifetime cigarette smoking	Never	1–29 day times	≥ 30 times
Current alternative tobacco products use	No	1 product	≥ 2 products
Frequency of alcohol consumption	Never for every type of alcoholic beverage	< 1 a month for any type of alcoholic beverage	≥ 1 a month for any type of alcoholic beverage
Number of alcoholic drinks per typical event*	Never drink	1 drink	≥ 2 drinks
Lifetime drunkenness*	Never	1 time	≥ 2 times
Frequency of binge drinking in past year*	Never	≤ 1 time a month	≥ 2 times a month
Lifetime illicit drug use*	Never for all drugs	Used any drug 1 time	Used ≥2 drugs or any drug ≥2 times
Lifetime cannabis use*	Never	1–5 times	≥ 6 times
Lifetime sexual history	No experience of sexual intercourse	Had sexual intercourse and always used contraception	Had sexual intercourse without contraception ≥1 time
Helmet use while riding bicycle	Never ride bike or always used helmet	Wore helmet sometimes or most of time	Never wore helmet
Caffeinated beverage consumption	Never	≤ 1 day week	≥ 2 days a week

*not included in analysis of grade 6–8 students because items were not included in their survey

### Confounding variables

Confounding variables included age, gender, self-rated family affluence as measure of socioeconomic status, ethnicity, and body mass index z-scores based on WHO growth references (de Onis et al., 2007). These variables were selected because they are correlates of concussion (based on an unpublished literature review we performed) and because they were assessed in the HBSC survey.

### Statistical analysis

Statistical analyses were performed using SPSS version 24 (IBM, Armonk, New York, USA). Variance estimates were adjusted to account for clustering by classroom. Descriptive statistics were used to describe the sample. Chi-square analyses determined if concussion prevalence varied across MRB quartiles. Logistic regression determined whether the relative odds of concussion differed across MRB quartiles. A p trend test determined if there was a dose-response relationship. Regression models controlled for confounding variables.

## Results

**Fig. 1:**
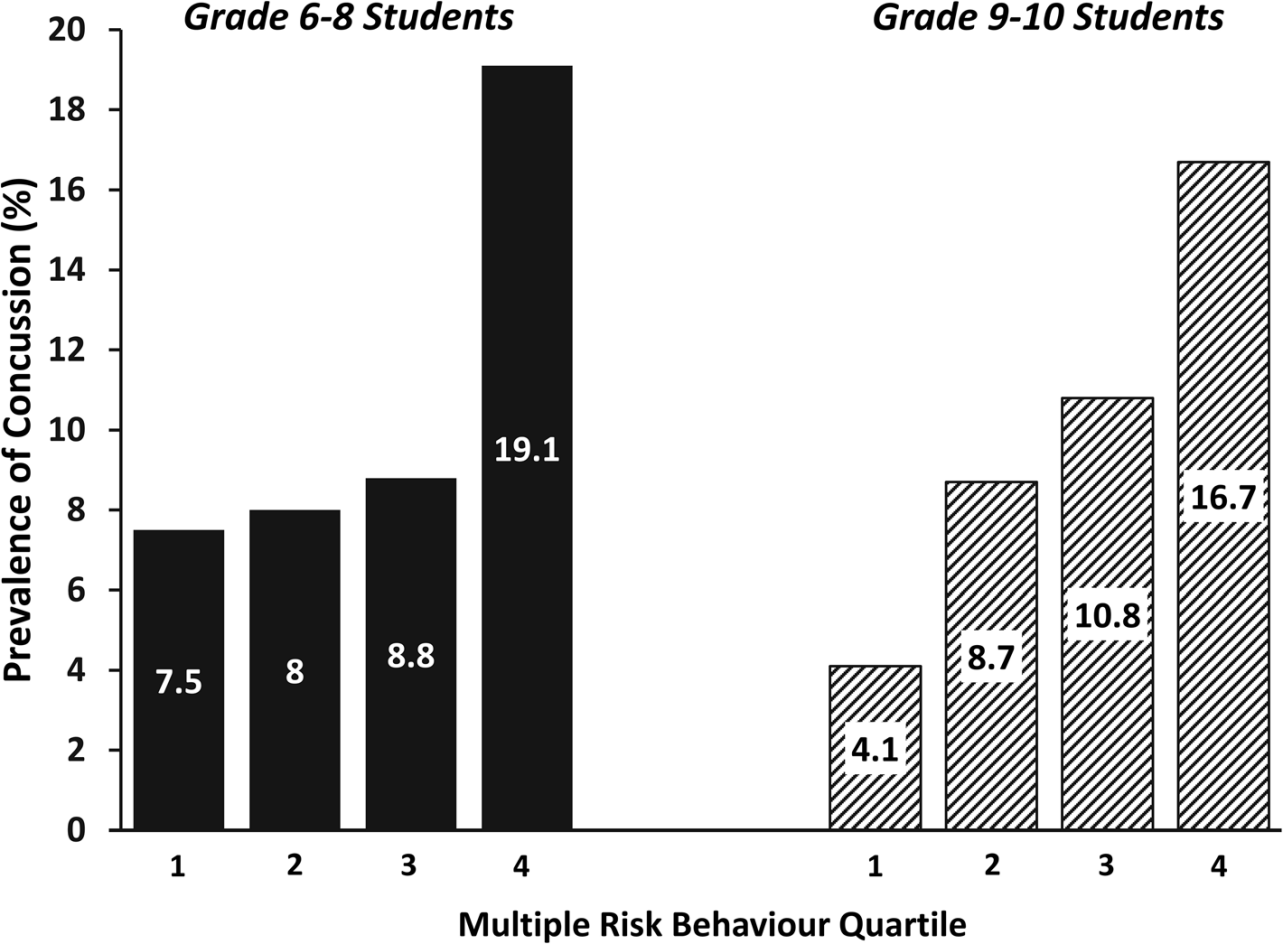
Proportion of grade 6–8 students (solid bars) and grade 9–10 students (hatched bars) who reported that they had a medically diagnosed concussion within the past year according to their level of engagement in multiple risk behaviours

Descriptive characteristics are in Table 2. One thousand nine hundred seventy-six participants were in grades 6–8 and 1086 were in grades 9–10. There were slightly more females than males. The majority were Caucasian (71.7%) and identified their family affluence as average or quite well-off (87.3%). As shown in Fig. 1, the prevalence of concussion increased significantly (*p* < .001) across MRB quartiles within both grades 6–8 and 9–10 students. The results of the logistic regression analyses are in Table 3. In grade 6–8 students, the relative odds of concussion was not significantly higher (*p* > 0.05) for the second or third MRB quartile by comparison to the first quartile. However, by comparison to quartile 1, the relative odds of concussion in was 2.94 (95% CI: 1.90, 4.56) in quartile 4 (*p* < .01). In grade 9–10 students, a dose-response relationship was observed between MRB and concussion (p_trend_ < 0.001). Quartile 4 exhibited relative odds of 4.67 (95% CI: 2.33, 9.35) by comparison to quartile 1 (*p* < .001).

**Table 2:** Descriptive characteristics of study sample

	Total Sample (*n* = 3059)	Grade 6–8 (*n* = 1976)	Grade 9–10 (*n* = 1083)
Gender			
Male	1449 (47.4)	959 (48.5)	490 (45.2)
Female	1610 (52.6)	1017 (51.5)	593 (54.8)
No. concussions in past year			
0	2732 (89.3)	1760 (89.1)	972 (89.8)
1	268 (8.8)	178 (9.0)	90 (8.3)
≥ 2	59 (1.9)	38 (1.9)	21 (1.9)
Ethnicity			
White	2194 (71.7)	1429 (72.3)	765 (70.6)
Asian	355 (11.6)	204 (10.3)	151 (13.9)
African American	31 (1.0)	19 (1.0)	12 (1.1)
Other or multi-ethnic	479 (15.7)	324 (16.4)	155 (14.3)
Perceived family wealth			
Not at all or not very well-off	304 (9.9)	185 (9.4)	119 (11.0)
Average or quite well-off	2096 (68.5)	1334 (67.5)	762 (70.4)
Very well-off	659 (21.5)	457 (23.1)	202 (18.7)

Data presented as n (%)

**Table 3:** Relative odds of concussion according to multiple risk behaviour quartile within grade 6–8 students and grade 9–10 students

Multiple risk behaviour quartile	Unadjusted model, OR (95% CI)	Adjusted model, ^*^ OR (95% CI)
Grade 6–8 students (n = 1976)		
Quartile 1 (referent)	1.00	1.00
Quartile 2	1.19 (0.80, 1.83)	1.10 (0.68, 1.77)
Quartile 3	1.31 (0.86, 1.99)	1.22 (0.76, 1.96)
Quartile 4	3.50 (2.40, 5.08)	2.94 (1.90, 4.56)
Grades 9–10 (n = 1083)		
Quartile 1 (referent)	1.00	1.00
Quartile 2	2.35 (1.16, 4.74)	2.21 (1.05, 4.65)
Quartile 3	2.94 (1.49, 5.82)	2.71 (1.32, 5.56)
Quartile 4	4.74 (2.47, 9.09)	4.67 (2.33, 9.35)

*OR*, Odds ratio; *CI*, Confidence interval

^*^Adjusted for age, gender, ethnicity, perceived family affluence, and body mass index z-score

## Discussion

This study provides a novel analysis of the relationship between MRB and concussion. The dose-response relationship between MRB and concussion observed in grade 9–10 students is consistent with previous studies that found dose-response relationships for adolescent injury based on level of engagement in MRB (Koven et al., 2005; Pickett et al., 2002a; Pickett et al., 2002b). In the grade 6–8 students, only the highest MRB quartile exhibited greater odds of concussion. These different grade-based patterns may reflect the different risky behaviours included in the composite MRB scores. It is possible that the risky behaviours not included in the survey administered to grades 6–8 students (e.g., unprotected sex, drug use) are more strongly associated with concussion. Alternatively, the relationship between MRB and concussion may become strengthened as students get older and engage in more severe risky behaviours more frequently.

Our results suggest that engagement in MRB may be a correlate of adolescent concussion. We speculate that the mechanisms explaining the link between some of the MRB (e.g., physical fighting, not wearing a helmet while riding a bicycle) and concussion reflect direct mechanisms (e.g., trauma caused by being punched in the face, hitting head when falling off bicycle, etc.). Conversely, we speculate that indirect mechanisms explain the link between concussion and many of the other MRB (e.g., smoking, alcohol, and drug use). For these risky behaviours, the association may be explained by the fact that adolescents who engage in these MRB are risk takers who make decisions and behave in ways that put themselves at heightened risk for concussion (e.g., behave recklessly when riding a bicycle).

Pediatric concussion prevention has focused on protective equipment and rule modification in sport (Benson et al., 2009; Cusimano et al., 2013). The MRB assessment strategy described in this study may be useful for identifying adolescents at increased concussion risk. Future studies should address whether behavioural interventions designed to decrease engagement in MRB may be useful for reducing concussion in youth.

There are several limitations to our study. Most notably, the cross-sectional design precludes us from knowing the direction of the relationship between MRB and concussion. This study only considered medically diagnosed concussions that were self-reported by students. The self-reported concussion and MRB items are vulnerable to recall biases that likely led to measurement error and conservative odds ratio estimates. Finally, a large number of participants were removed from the analysis because of missing data.

## Conclusions

Risk for concussion was significantly elevated among adolescents with the highest level of MRB engagement. This study highlights the need to appreciate concussion as a broader public health concern with modifiable behavioural risk factors. Future studies should consider behavioural interventions designed to decrease adolescent engagement in multiple risk behaviours, in order to reduce concussion in youth.

## Data Availability

The data for the HBSC are not publicly available due to ethics and privacy concerns.

## Abbreviations

HBSC: Health Behaviour in School-Aged Children survey
MRB: multiple risk behaviours
